# RNA-silencing nanoprobes for effective activation and dynamic imaging of neural stem cell differentiation

**DOI:** 10.7150/thno.35032

**Published:** 2019-07-09

**Authors:** Ruili Zhang, Zhe Wang, Zuo Yang, Linlin Wang, Zhongdi Wang, Baihang Chen, Zhongliang Wang, Jie Tian

**Affiliations:** 1 Engineering Research Center of Molecular- and Neuro-imaging of ministry of education, School of Life Science and Technology, Xidian University, Xi'an, Shaanxi, 710126 China.; 2 Key Laboratory of Molecular Imaging, Institute of Automation, Chinese Academy of Sciences, Beijing, 100190, China.; 3 Beijing Advanced Innovation Center for Big Data-Based Precision Medicine, School of Medicine, Beihang University, Beijing, 100191, China; 4 Laboratory of Molecular Imaging and Nanomedicine, National Institute of Biomedical Imaging and Bioengineering, National Institutes of Health, Bethesda, Maryland, 20892 USA

**Keywords:** gold nanoparticle, gene silencing, imaging, drug delivery, neural stem cell

## Abstract

To achieve the clinical potential of neural stem cells (NSCs), it is crucial to activate NSC differentiation into neurons and simultaneously monitor the process of NSC differentiation. However, there are many challenges associated with regulating and tracking NSC differentiation.

**Methods**: We developed a redox-responsive multifunctional nanocomplex with a disulfide bond—cvNC—for the delivery of siRNAs to induce NSC differentiation through sequence-specific RNA interference (RNAi) and real-time imaging of sequential mRNA expression during differentiation. The stability and specificity of cvNCs were studied *in vitro*. Controlled release of siRNA, gene silencing efficiency, as well as real-time imaging of cvNCs on *Tubb3* and *Fox3* mRNAs during NSC differentiation were evaluated.

**Results**: The introduction of a redox-sensitive disulfide bond not only ensures the remarkable performance of cvNC, such as high stability, controlled siRNA release, and enhanced gene silencing efficiency, but also effectively stimulates NSC differentiation into neurons. More importantly, the cvNC can track NSC differentiation in real-time by monitoring the sequential expression of mRNAs.

**Conclusion**: Our study indicates that cvNC can serve as a robust system for exploring NSCs differentiation process as well as other biological events in living cells.

## Introduction

Neural stem cells (NSCs) have attracted increased attention over the past decades by virtue of their self-renewal and multipotency, the ability to differentiate into all types of neural cells—neurons, astrocytes, and oligodendrocytes 1. These unique characteristics not only offer a powerful tool for basic research but also make NSCs extremely valuable in regenerative therapies for diseases and injuries of the central nervous system (CNS) 2-5. Effectively directing NSC differentiation into a desired cell type, like regenerated neurons, has become particularly important to achieve the clinical potential of NSCs 6.

In this regard, the most widely used strategy is to modulate specific signaling cascades in the cells to eventually induce the expression of neuronal genes in the NSCs. As a sequence-specific mechanism for genetic regulation, RNA interference (RNAi) that can silence almost all genes, holds unlimited potential in treating CNS diseases 7, 8. Small interfering RNAs (siRNAs) have been developed to target and degrade complementary messenger RNAs (mRNAs) to suppress target protein expression. Despite the immense potential of siRNA to silence important genes responsible for various diseases, safe and efficient delivery of siRNAs remains challenging due to the intrinsic deficiencies of siRNA 5, 9-14. For example, unmodified siRNA is easily degraded by nucleases and does not readily cross membranes to enter cells, impairing the efficacy of siRNAs. To successfully control NSC differentiation using RNAi, siRNAs must be brought to its site of action without degradation. To improve siRNA bioavailability, covalent or noncovalent interactions have been utilized to fabricate several siRNA delivery systems 15-21. Although emphasis is often placed on the importance of cellular uptake of siRNA delivery systems, controlled release is also crucial to keep siRNA concentration within its action window and improve gene silencing efficiency. Stimuli-responsive delivery systems have emerged as a promising option to achieve site-specific release of siRNA and to deliver the required concentration at the target site 22, 23. Among various stimuli-sensitive linkages, disulfide bond possessing redox sensitivity offers additional advantages as it preserves adequate stability in the extracellular environment and undergoes rapid cleavage when exposed to the more reducing intracellular compartment 24. Thus, redox-responsive delivery systems with crosslinked disulfide bonds may minimize burst-release and significantly enhance the bioavailability of siRNAs.

In addition to the challenge of controlling differentiation, a further but more complicated challenge in NSC regenerative therapy is monitoring the differentiation of NSCs in real-time 25. When it comes to monitoring, intracellular imaging of mRNA expression during NSC differentiation is very instructive, as specific mRNAs are expressed at specific stages of neuronal development 26, suggesting that the dynamics of neuron-specific mRNA expression reflects neuron generation, progression, and interaction with the environment. Therefore, it is reasonable to speculate that mRNAs are ideal for real-time tracking of NSC differentiation.

**Scheme 1:**
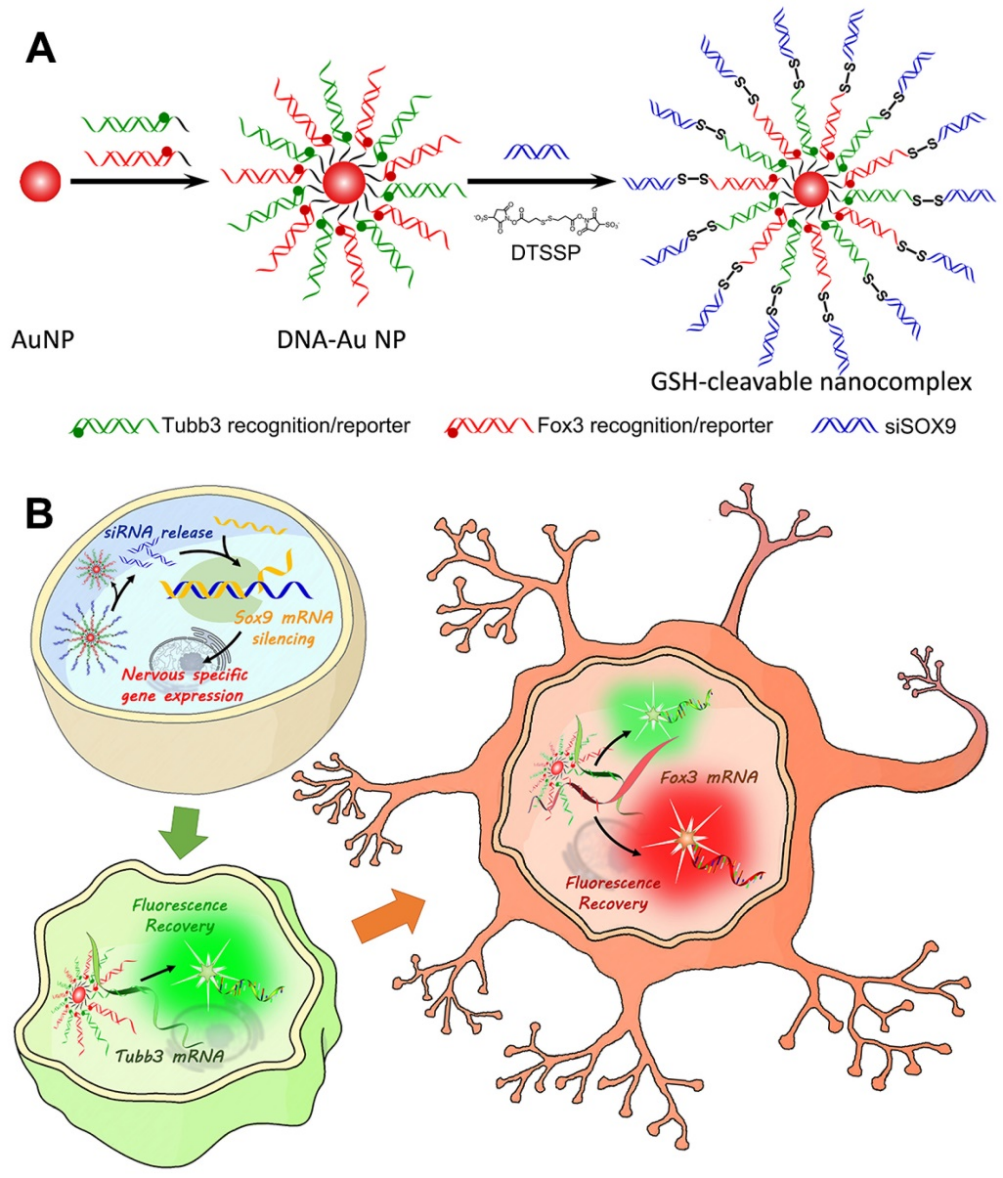
Design and properties of GSH-cleavable nanocomplexes (cvNCs). (A) Synthesis of cvNCs. (B) cvNCs trigger cascade activation and allow imaging of the dynamic differentiation of neural stem cells (NSCs).

Herein, taking advantage of the disulfide linkage, we developed a redox-responsive multifunctional platform for delivering siRNAs to direct NSC differentiation and simultaneous dynamic imaging of sequential mRNA expression in differentiating neurons (Scheme 1). We believe our system is robust and reliable based on the following considerations. 1) As mentioned above, inducing the expression of neuronal genes in NSCs is a common strategy to induce NSC differentiation into neurons. So, modulating gene expression using RNAi is more potent than only using antisense effect of oligonucleotides. 2) SOX9 protein is reported to actively inhibit neuronal gene expression inducing majority of the NSCs to differentiate into astrocytes (glial cells) 27, 28. Therefore, our strategy of silencing SOX9 expression using siRNA would significantly increase the percentage of NSCs adopting a neuronal fate. 3) Owing to their attractive features, such as low cellular toxicity and easy surface functionalization, gold nanoparticles (AuNPs) were employed to deliver siRNA against SOX9 (siSOX9) and enable efficient self-delivery of siRNA with enhanced stability. 4) Considering that the disulfide bond can be cleaved by reducing agents like glutathione (GSH) 24, we formulated a reduction-sensitive disulfide bond to bridge siSOX9 and DNA-AuNPs for achieving accurate intracellular release of siSOX9 and to enhance its bioavailability. It is worth mentioning that the disulfide linkage plays an essential role in our design. We fabricated another siSOX9 delivery system without the disulfide bond as a non-cleavable control (termed as non-cvNC, Figure S1), and we only achieved a weak RNAi effect. 5) Two DNA sequences hybridized with different fluorophore reporters were designed to recognize two neuron markers—TuJ1 encoded by *Tubb3* and NeuN encoded by *Fox3*—which are sequentially expressed during NSC differentiation from progenitor cells into neurons to allow dynamic imaging of siRNA-mediated mRNA expression through fluorescence enhancement upon sequence-specific competitive binding.

## Materials and Methods

### Reagents and instrumentation

All solvents and starting materials were purchased from TCI Shanghai or Sigma-Aldrich and used without further purification. Transmission electron microscopy (TEM) was performed using a JEOL 200X operated at 200 kV. UV-vis absorption spectra were obtained using a SHIMADZU UV-1701 spectrophotometer. Dynamic Light Scattering (DLS) analysis was carried out using a Zetasizer Nano ZS.

A detailed description of the synthesis and characterization of all compounds is provided in the Supplementary Materials.

### Synthesis of GSH-cleavable nanocomplexes

Citrate-stabilized gold nanoparticles (15 ± 0.6 nm) were prepared as previously reported 29. Two dithiol-modified recognition oligonucleotides were mixed separately with corresponding reporter oligonucleotides (1:1.2 of molar ratio) in phosphate buffered saline (PBS; pH 7.4, 10 mM phosphate, 27 mM KCl, 5 mM MgCl_2_, and 138 mM NaCl), heated up to 75 °C and maintained for 30 min. It was then slowly cooled down to room temperature and stored in the dark for at least 12 h to allow hybridization. These DNA duplexes were mixed together and added to a gold nanoparticle solution (2 nM) at final concentrations of 360 nM Alexa 488-*Tubb3* DNA duplex and 380 nM Cy3-*Fox3* DNA duplex, and shaken overnight, after which 1.5 M sodium chloride was added gradually to bring the NaCl concentration to 0.3 M. The solution was further shaken for 8 h. The resulting DNA-AuNPs were purified by centrifugation (12000 rpm, 10 min, three times), and was resuspended in PBS.

DNA-Au NPs (0.05 nmol in PBS; pH 8.0) was mixed with 10 nmol DTBP, shaken for 1 h in the dark, and purified by centrifugation (12000 rpm, 10 min, three times), and was resuspended in PBS (pH 8.0). After that, 10 nmol siSOX9 was added to the above solution, shaken for 1 h in the dark, and purified by centrifugation (12000 rpm, 10 min, three times), and was resuspended in PBS. Particles were sterilized using a 0.2 µm acetate syringe filter.

### Controlled release of siSOX9 *in vitro*

Samples of cvNCs, siSOX9, cvNCs and GSH, non-cvNCs, non-cvNCs and GSH were analyzed by agarose gel electrophoresis, which was performed at 90 V for 45 min using a 4% agarose gel in 1× TBE buffer (89 mM tris(hydroxymethyl)aminomethane, 2 mM ethylenediamine tetraacetic acid, and 89 mM boric acid, pH 8.0). The DNA bands were stained with GeneGreen Nucleic Acid Dye and visualized by UV illumination.

### Controlled release of siSOX9 in Cells

Cells were plated in a 35 mm cover glass-bottom dish (MatTek Corp., USA) and incubated overnight before the experiment. Cells were washed twice with 1× PBS and then incubated with cvNCs or non-cvNCs at the desired concentration at 37 °C and 5% CO_2_. After incubation, cells were washed twice with 1× PBS, suspended in 1× PBS and observed by confocal microscopy.

### RNA extraction and qRT-PCR analysis

RNA samples were extracted from NSCs using a TriPure isolation reagent (Roche). To prevent DNA contamination, total RNA was treated with RNase-free DNase II (Invitrogen). Total RNA samples (1 µg per reaction) were reverse transcribed into cDNAs using SuperScriptTM First-Strand Synthesis System (Invitrogen). The cDNAs were used as templates for quantitative real-time PCR. The amplification reactions were performed using the Fast SYBR Green Master Mix on a StepOne Plus real-time PCR system (Applied Biosystems, Foster City, CA) according to the manufacturer's protocol. The Primers for SOX9 gene were 5′-AGGAAGCTGGCAGACCAGTACC-3′ (Forward) and 5′-TCTCTTCTCGCTCTCGTTCA-3′ (Reverse). The human glyceraldehyde-3-phosphate dehydrogenase (GAPDH) was used as an internal control. The GAPDH primers were 5′-GGTCTCCTCTGACTTCAACA-3′ (Forward) and 5′-AGCCAAATTCGTTGTCATAC-3′ (Reverse).

### Specificity experiments of cvNCs

*Tubb3*-targeted or *Fox3*-targeted DNA-Au NPs of 1 nM in PBS buffer were treated with different DNA targets of 200 nM, including completely complementary DNA targets, survivin, β-actin, GAPDH, CXCR4, and Nestin. After 1-h incubation at 37 °C, the fluorescence of Alexa 488 was excited at 490 nm and measured at 530 nm (range from 500 to 700 nm), and the fluorescence of Cy3 was excited at 550 nm and measured at 570 nm (range from 550 to 700 nm). All experiments were repeated at least three times.

### Nuclease assay for cvNCs

DNA-AuNPs were diluted to a concentration of 1 nM in buffer (10 mM PBS, pH 7.4, 2 mM MgCl_2_, 1 mM CaCl_2_ and 50 mg/L Bovine Serum Albumin). DNase I (Amplified grade, Invitrogen) at a final concentration of 1 U/L was added immediately before reading after allowing the samples to equilibrate for 10 min. Molecular beacons were tested in an analogous manner at a concentration of 50 nM. The fluorescence was monitored for 24 h and was measured at predetermined intervals during this period.

### Real-time imaging of cvNCs on *Tubb3* and *Fox3* mRNAs in differentiated NSCs

NSCs were cultured in 8-well Lab-Tech chamber slides (NUNC, Thermo Scientific) for 24 h prior to the experiment. cvNCs were then incubated with the NSCs. The first day after incubation, 0.5 µL Hoechst 33342 stock solution was added into each well for live cell nucleus staining 30 min before imaging. A mark was made on the chamber to ensure that the same cell colony was imaged for 8 days. On the second day before imaging, cells were washed with HBSS solution (pH = 7.4) twice, and culture media was renewed. Culture medium was changed every other day until day 10, and imaging was performed every day. Hoechst 33342 was added whenever necessary to identify the nucleus and the colony of interest. For negative control, cvNCs were incubated with a scrambled sequence, or non-cvNCs and imaged for 10 days using the same protocol.

## Results and Discussion

### Design and preparation of GSH-cleavable nanocomplex

To construct the redox-responsive multifunctional platform (GSH-cleavable nanocomplex, termed cvNC, Scheme 1A), DNA-Au NPs were first fabricated by modifying pure AuNPs with a dense layer of recognizing DNA oligonucleotides, consisting of a 6-base-pair (bp) adenine (A6) spacer and a 21-bp fragment complementary to the corresponding nucleotide sequence (*Tubb3*: 228-248; *Fox3*: 1349-1369). *Tubb3* is a marker seen in the earliest stages of neuronal development, while *Fox3* is specifically expressed in post-mitotic neurons. To visualize the process of NSC differentiation, different fluorophore-capped reporter sequences (*Tubb3*: Alexa488; *Fox3*: Cy3) were hybridized with the 12 bp fragment of the corresponding recognizing sequence (Table S1). Most importantly, the termini of the recognizing oligonucleotides of DNA-AuNPs were further linked with siSOX9 via cleavable disulfide bond to ensure the controlled release of siSOX9 inside the cells. In addition, DNA-AuNPs directly linked with siSOX9 without disulfide bond were fabricated as non-cvNCs (Figure S1). cvNCs are stable in the extracellular environments, and the fluorophores are initially quenched by AuNPs through fluorescence resonance energy transfer (FRET), whereas upon cellular uptake, the disulfide linkage is cleaved by GSH to release siSOX9, which activates the RNAi pathway and promotes neuron-specific gene expression (Scheme 1B). With the development of progenitor cells differentiating to neurons, the target mRNAs expressed sequentially at specific stages are identified by the recognizing oligonucleotides on DNA-AuNPs, which hybridize with the target mRNAs boosting the release of the fluorophore reporter from the cvNCs enabling dynamic imaging of mRNA expression (Scheme 1B).

**Figure 1:**
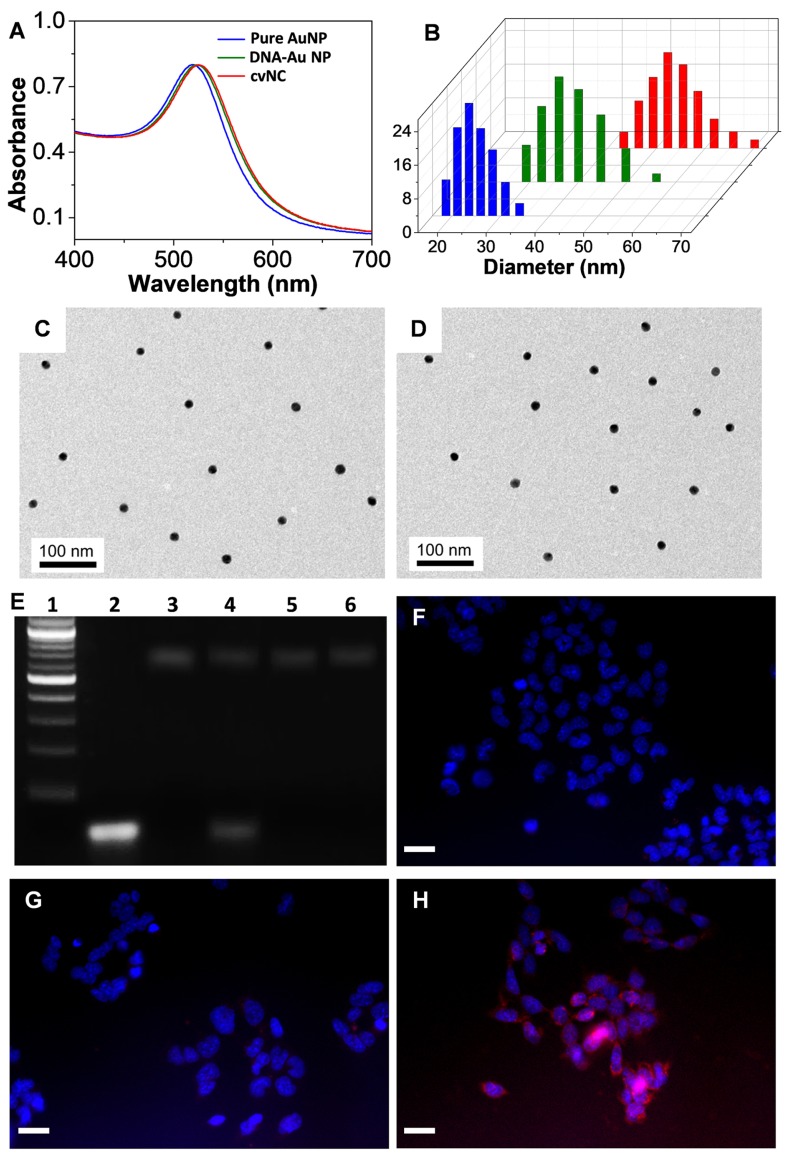
Characterization of cvNCs and evaluation of siSOX9 release. UV-Vis spectra (A) and dynamic light scattering analysis (B) of pure AuNPs (blue), DNA-AuNPs (green), and cvNCs (red). TEM images of pure AuNPs (C) and cvNCs (D). (E) Agarose gel electrophoresis of Marker (Lane 1), siSOX9 (Lane 2), cvNCs (Lane 3), cvNCs with GSH (Lane 4), non-cvNCs (Lane 5), and non-cvNCs with GSH (Lane 6). Fluorescence microscopy images of NSCs untreated (F) and treated with non-cvNCs (G), and cvNCs (H), respectively. Blue: nuclei stained with Hoechst 33342, Red: Cy5-siSOX9. Scale bar: 40 µm.

To validate the functionality of cvNCs, we first characterized the formation of cvNCs. Compared to the pure AuNPs, the surface plasmon resonance (SPR) band of cvNCs exhibited a 5 nm red-shift, resulting from the functionalization of DNA and siSOX9 molecules on the AuNP surface (Figure 1A). This successful loading of DNA and siSOX9 molecules is further supported by results from DLS measurements (Figure 1B), which showed that the average hydrodynamic radius of cvNCs (55 nm) increased by 30 nm, and the surface charge of cvNCs shifted more to the negative compared to that of the pure AuNPs (-16 ± 2.0 mV vs. -33 ± 2.6 mV) after surface functionalization. In addition, the resulting cvNCs showed high monodispersity and uniform particle size with a diameter of 15 ± 0.5 nm (Figures 1C and D). On average, cvNC was calculated to carry 24 ± 1 *Tubb3* recognition/reporter duplexes, 26 ± 2 *Fox3* recognition/reporter duplexes, and 41 ± 2 siSOX9 (Figure S2).

### Controlled release of siSOX9 *in vitro* and cells

In our design, the disulfide linkage is critical for achieving controlled release of siRNA. Therefore, we next evaluated the GSH-responsiveness of cvNCs and subsequent siRNA release by agarose gel electrophoresis. As shown in Figure 1E, cvNCs exhibit good stability and do not show measurable cleavage activity in the absence of GSH. Upon addition of GSH, however, cvNCs can be cleaved to produce a fragment, which matches the size of siSOX9, suggesting that cvNCs mediate site-specific siRNA release. As a result, the cleavage site should be the predicted position where the disulfide bond linked siRNA and recognition DNA on the surface of AuNPs. In contrast, non-cvNCs do not show any GSH-induced cleavage. These results demonstrate that cvNCs permit GSH-triggered siRNA release. Next, we evaluated whether cvNCs could respond to intracellular GSH and achieve intracellular siRNA release in a self-activating fashion. To answer this question, the siSOX9 sequence on cvNCs was labeled with Cy5, which was initially quenched by the wide-spectrum AuNPs. However, once siSOX9 is released, the fluorescence of Cy5 would be recovered quickly, allowing fluorescence imaging of cells. As depicted in Figures 1F-H, NSCs treated with cvNCs displayed a strong fluorescence, while cells treated with non-cvNCs did not reveal any obvious fluorescence. This result validates the cleavage of the disulfide bonds between recognizing oligonucleotides of AuNPs and siSOX9 by GSH within cells, thereby promoting the recovery of Cy5 fluorescence.

### Cytotoxicity of cvNCs to NSCs

Before intracellular siRNA release experiment, the cytotoxicity of cvNCs to NSCs was evaluated using a 3-(4,5-dimethylthiazol-2-yl)-2,5-diphenyltetrazolium bromide (MTT) assay. As shown in Figure S3, there was no detectable cytotoxicity after a 24 h incubation with different concentrations of cvNCs, suggesting good biocompatibility of the cvNCs.

### Intracellular activity of cvNCs in NSCs

**Figure 2:**
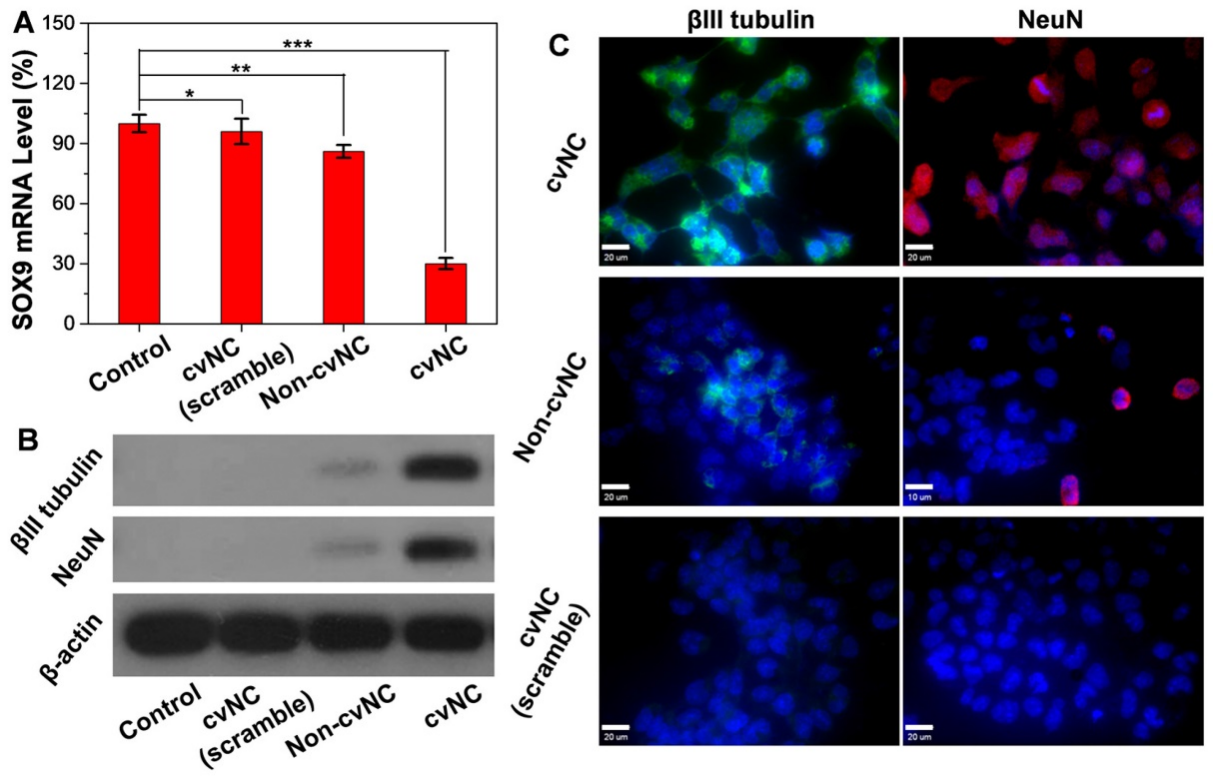
Intracellular activity of cvNCs in the NSCs. (A) qRT-PCR analyses of SOX9 mRNA expression and (B) western blot analyses of βIII tubulin and NeuN expression in NSCs treated with cvNC or controls for 8 days. Student's t test, *: *P* > 0.41, **: *P* < 0.013, ***: *P* = 0.000018. (C) Immunofluorescence staining of βIII tubulin (green) and NeuN (red) in NSCs treated with cvNC without reporters or control cvNCs at day 8. Scale bar: 20 µm.

Inspired by the controlled release of siSOX9, we further tested the intracellular activity of cvNCs in NSCs by quantitative real-time polymerase chain reaction (qRT-PCR) and western blot assays. As shown in Figure 2A, cvNCs significantly down-regulated the expression of SOX9, resulting in ~70% decrease in SOX9 transcription. On the other hand, non-cvNCs induced only a slight decrease in SOX9 mRNA level. cvNCs with scrambled sequence barely displayed gene silencing effects. Western blot results were consistent with the qRT-PCR results. The cells treated with cvNCs alone showed a significant increase in *Tubb3*-coding for βIII tubulin and *Fox3*-coding for NeuN (Figure 2B). Such impressive gene knockdown capability of cvNCs can be attributed to the disulfide bond, which allowed the site-specific release of siRNA inside the cell for more efficient formation of RNA-induced silencing complex (RISC) leading to the cleavage of target SOX9 mRNA. However, the densely packed DNA molecules on non-cvNCs probably blocked the formation of RISC due to steric hindrance and repulsive Coulomb interactions, resulting in a poor RNAi efficacy. Therefore, the GSH-responsive feature of cvNCs not only achieved the controlled intracellular release of siSOX9 but also contributes to the striking gene silencing effect, which is favorable for promoting NSC differentiation into neurons.

To further explore the cvNC's potency to induce NSC differentiation, we transfected NE-4C NSCs with cvNCs or controls non-cvNCs and observed the sequential expression of two neuronal markers (βIII tubulin and NeuN) by immunofluorescence staining. As shown in Figure 2C, the cvNCs-treated cells displayed efficient intracellular distribution of βIII tubulin (green) and NeuN (red), which strongly support the effectiveness of cvNCs in stimulating NSC differentiation over long-term induction. In contrast, non-cvNCs only achieved minimal βIII tubulin and NeuN expression. Neither βIII tubulin nor NeuN expression was observed when cvNCs with scrambled sequence were used. This is consistent with our results in Figures 2A and B, showing the advantage of disulfide linkage for high-efficiency delivery of siRNA and subsequent knockdown of gene expression. Collectively, our cvNCs could achieve GSH-triggered intracellular release of siSOX9, promoting NSC differentiation into neurons.

### Specificity and stability of cvNCs as imaging probes

**Figure 3:**
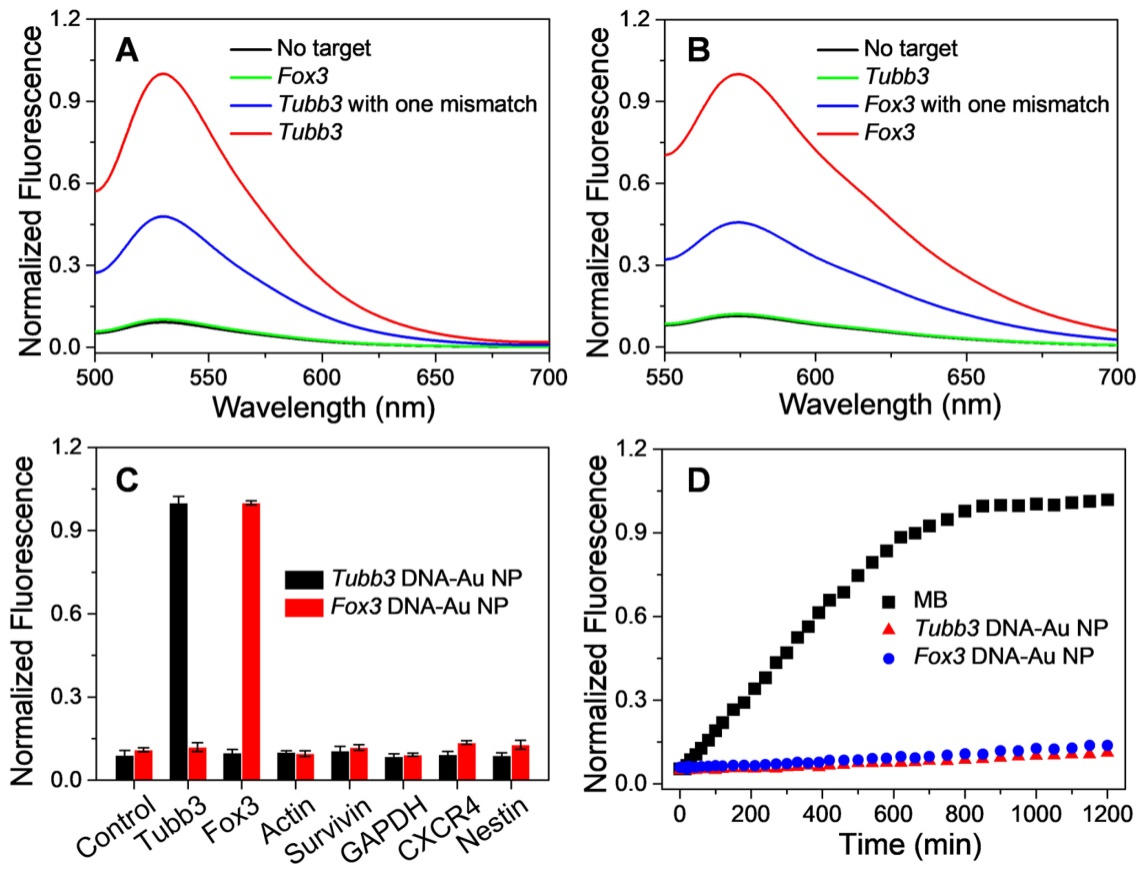
Sensitivity and specificity of DNA-Au NPs to DNA targets. Fluorescence spectra of *Tubb3*-targeted DNA-AuNPs (A) and *Fox3*-targeted DNA-AuNPs (B) in the presence of no target (black line), complementary target (red line), cross-talk target (green line), or complementary target with one mismatch (blue line). (C) Specificity of DNA-AuNPs to DNA targets. (D) Enzymatic stability study of molecular beacons (black), *Tubb3*-targeted DNA-AuNPs (red), and *Fox3*-targeted DNA-Au NPs (blue) treated with DNase I.

In addition to controlling the differentiation of NSCs, another issue which merits our attention is how to monitor the differentiation of NSCs in real-time. Accordingly, we specially designed fluorescence reporter sequences in the structure of cvNCs, aiming to visualize the differentiation process dynamically. With regard to imaging, specificity and sensitivity are the two important indicators to determine the imaging performance of the probe. Thus, we carefully tested the binding specificity of cvNCs toward the DNA targets *in vitro*. As depicted in Figure 3A and B, in distinct contrast with the extremely weak fluorescence without the DNA target, the fluorescence intensity of DNA-AuNPs was significantly increased by over nine-fold in the presence of a completely complementary DNA target for both Tubb3 and Fox3 without any cross-talk response. Moreover, DNA-AuNPs are able to further differentiate a completely matched DNA target from mismatched DNA target even with a single-base mutation, which showed about two-fold lower fluorescence intensity. Furthermore, in sharp contrast with low specificity toward off-targets, DNA-AuNPs present excellent specificity and sensitivity to *Tubb3* or *Fox3* target (Figure 3C). The stability of DNA-AuNPs and molecular beacon (MB) against enzyme degradation were comparatively investigated by monitoring the fluorescence change after incubation with DNase I endonuclease. As shown in Figure 3D, the fluorescence from MB was intensified substantially within 10 h, while DNA-AuNPs only yielded a slight increase in fluorescence during 20 h of incubation, suggestive of the enhanced stability of DNA-AuNPs to enzymatic degradation. The fluorescence response is fast (<20 s) and highly associated with DNA target (Figure S4), demonstrating that DNA-Au NPs are suitable for real-time mRNA imaging in living cells. It is the superior specificity, sensitivity, and stability of DNA-AuNPs *in vitro* that encouraged us to examine its performance as a fluorescence probe to monitor NSC differentiation.

### Self-activation and dynamic imaging of mRNA sequential expression during NSC differentiation

**Figure 4:**
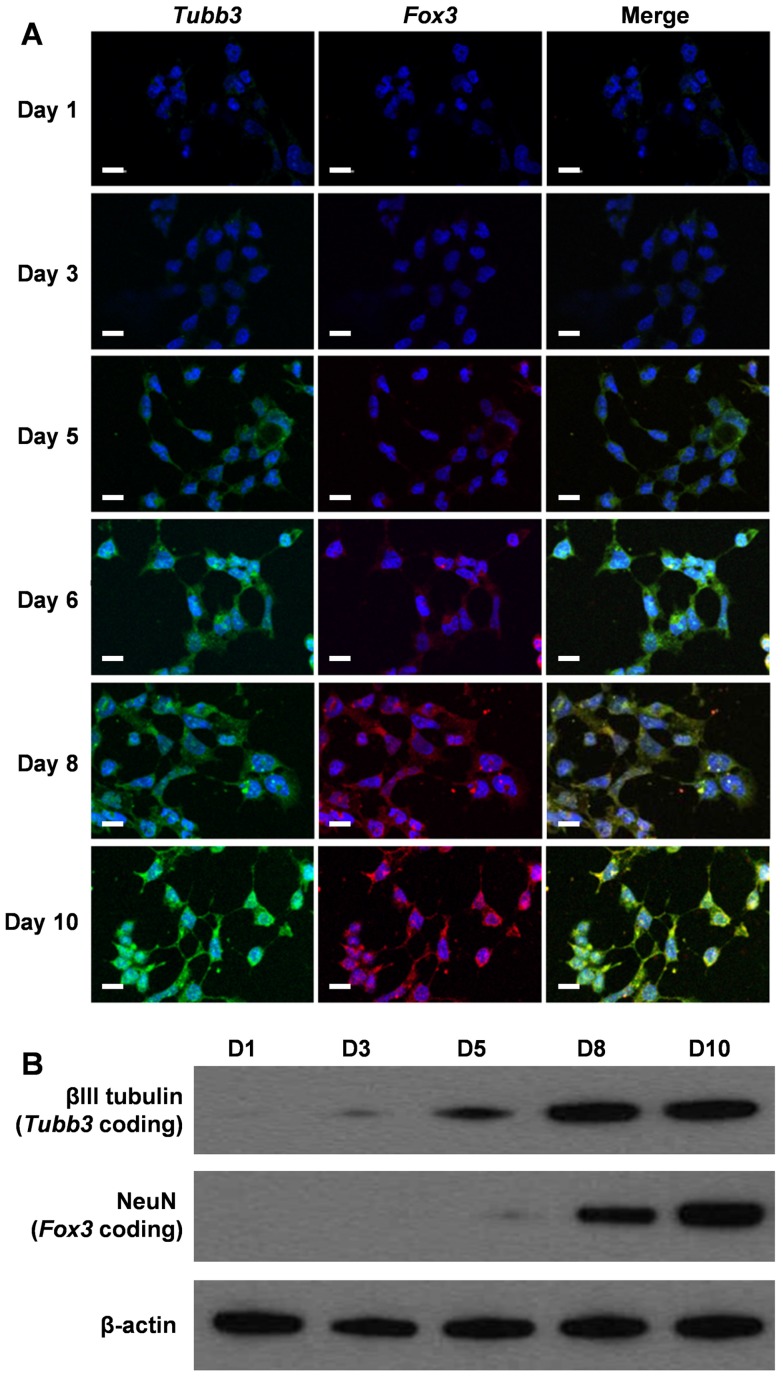
(A) Self-activating and spatiotemporal imaging of *Tubb3* and *Fox3* mRNA sequential expressions during NSC differentiation in living cells. Green: *Tubb3* mRNA; red: *Fox3* mRNA; blue: nuclei stained with Hoechst 33342. Scale bar: 20 µm. (B) Western blot analysis of βIII tubulin and NeuN at different stages of NSC differentiation

To assess whether our cvNCs could track the differentiation process of NSCs spatiotemporally, we activated NSC differentiation using cvNCs and monitored the sequential expression of mRNAs by examining the generation of fluorescence signals. Fluorescence microscopy images in Figure 4A clearly show that *Tubb3* mRNA expression was observed on day 3, which was about 2 days earlier than *Fox3* mRNA expression. Both markers peak expression levels on day 10. These results agree with the intrinsic biological functions of *Tubb3* and *Fox3*, which are present in the earliest stage of neuronal development and mature neurons, respectively. Undoubtedly, cvNCs can not only stimulate NSCs differentiation into neurons efficiently but also allows the dynamic monitoring of the sequential expression of *Tubb3* and *Fox3* mRNA with high sensitivity and specificity. Flow cytometry results (Figure S5) further confirmed our conclusion. In contrast, non-cvNCs could hardly activate NSC differentiation, leading to a sporadic fluorescence signal distribution (Figure S6) over 10-day incubation. As expected, cvNCs with scrambled sequence failed to induce the generation of neurons (Figure S7). This difference can be ascribed to the difference in structures between cvNCs and non-cvNCs, i.e., the disulfide bond, which promotes the release of siSOX9 and thus inhibits the expression of SOX9 mRNA. In addition, results from western blot analysis (Figure 4B) were consistent with the fluorescence microscopy results and supported the sequential expression of mRNAs during cvNC-induced NSC differentiation.

## Conclusions

In summary, we present a redox-responsive multifunctional nanocomplex, cvNC, which can not only deliver siRNA to induce NSCs differentiation but also allows the real-time monitoring of the sequential mRNA expression during the differentiation. In addition to the advantages of AuNPs, such as low cellular toxicity, easy modification, and wide-spectrum quenching capability, the disulfide linkage plays a critical role in boosting the performance of cvNCs. Our results demonstrate that the GSH-triggered cleavage of disulfide bond not only enables the controlled release of the siRNA inside cells, avoiding adverse effects but also helps to preserve the target specificity of siRNA, which is a major consideration when siRNA is attached to nanocarriers through covalent interaction. It is this superiority of cvNC that facilitates significant suppression of SOX9 gene expression, thereby effectively stimulating NSC differentiation into neurons. At the same time, the cvNC's capability to specifically recognize the two neuronal markers *Tubb3* and *Fox3* also enables spatiotemporally tracking NSC differentiation through real-time imaging of sequential mRNA expression, which is an important challenge in NSC regenerative therapy. Our cvNC exhibits excellent intracellular stability, high targeting specificity, site-specific siRNA release, and high-efficiency gene silencing, as well as dynamic mRNA expression imaging capability. By exquisite sequence design based on various target genes, cvNC provides excellent opportunity to explore the complicated process of NSC differentiation and other similar biological processes in living cells.

## Supplementary Material

Supplementary experimental procedures, figures and table.

## References

[1] Gage FH, Temple S (2013). Neural stem cells: generating and regenerating the brain. Neuron.

[2] Wang Z, Wang Y, Wang Z, Zhao J, Gutkind JS, Srivatsan A (2015). Polymeric nanovehicle regulated spatiotemporal real-time imaging of the differentiation dynamics of transplanted neural stem cells after traumatic brain injury. ACS Nano.

[3] Goldman SA (2016). Stem and progenitor cell-based therapy of the central nervous system: hopes, hype, and wishful thinking. Cell Stem Cell.

[4] Ardhanareeswaran K, Mariani J, Coppola G, Abyzov A, Vaccarino FM (2017). Human induced pluripotent stem cells for modelling neurodevelopmental disorders. Nat Rev Neurol.

[5] Shah S, Solanki A, Sasmal PK, Lee KB (2013). Single vehicular delivery of siRNA and small molecules to control stem cell differentiation. J Am Chem Soc.

[6] Ziller MJ, Edri R, Yaffe Y, Donaghey J, Pop R (2015). Dissecting neural differentiation regulatory networks through epigenetic footprinting. Nature.

[7] Wilson RC, Doudna JA (2013). Molecular mechanisms of RNA interference. Annu Rev Biophys.

[8] Wittrup A, Lieberman J (2015). Knocking down disease: a progress report on siRNA therapeutics. Nat Rev Genet.

[9] Kanasty R, Dorkin JR, Vegas A, Anderson D (2013). Delivery materials for siRNA therapeutics. Nat Mater.

[10] Wang J, Mi P, Lin G, Wáng YX, Liu G, Chen X (2016). Imaging-guided delivery of RNAi for anticancer treatment. Adv Drug Deliv Rev.

[11] Qiao C, Yang J, Shen Q, Liu R, Li Y, Shi Y (2018). Traceable nanoparticles with dual targeting and ROS response for RNAi-based immunochemotherapy of intracranial glioblastoma treatment. Adv Mater.

[12] Li J, Yu X, Wang Y, Yuan Y, Xiao H, Cheng D (2014). A reduction and pH dual-sensitive polymeric vector for long-circulating and tumor-targeted siRNA delivery. Adv Mater.

[13] Wang Z, Wang Z, Liu D, Yan X, Wang F, Niu G (2014). Biomimetic RNA-silencing nanocomplexes: overcoming multidrug resistance in cancer cells. Angew Chem Int Ed.

[14] Lei Y, Tang L, Xie Y, Xianyu Y, Zhang L, Wang P (2017). Gold nanoclusters-assisted delivery of NGF siRNA for effective treatment of pancreatic cancer. Nat Commun.

[15] Xu X, Wu J, Liu Y, Saw PE, Tao W, Yu M (2017). Multifunctional envelope-type siRNA delivery nanoparticle platform for prostate cancer therapy. ACS Nano.

[16] Song WJ, Du JZ, Sun TM, Zhang PZ, Wang J (2010). Gold nanoparticles capped with polyethyleneimine for enhanced siRNA delivery. Small.

[17] Ho W, Zhang XQ, Xu X (2016). Biomaterials in siRNA delivery: a comprehensive review. Adv Healthc Mater.

[18] Guo X, Huang L (2012). Recent advances in nonviral vectors for gene delivery. Acc Chem Res.

[19] Li J, Yang Y, Huang L (2012). Calcium phosphate nanoparticles with an asymmetric lipid bilayer coating for siRNA delivery to the tumor. J Control Release.

[20] Ding Y, Jiang Z, Saha K, Kim CS, Kim ST, Landis RF, Rotello VM (2014). Gold nanoparticles for nucleic acid delivery. Mol Ther.

[21] Jensen SA, Day ES, Ko CH, Hurley LA, Luciano JP, Kouri FM (2013). Spherical nucleic acid nanoparticle conjugates as an RNAi-based therapy for glioblastoma. Sci Transl Med.

[22] Yin J, Lang T, Cun D, Zheng Z, Huang Y, Yin Q (2017). pH-sensitive nano-complexes overcome drug resistance and inhibit metastasis of breast cancer by silencing Akt expression. Theranostics.

[23] Kim HJ, Kim A, Miyata K, Kataoka K (2016). Recent progress in development of siRNA delivery vehicles for cancer therapy. Adv Drug Deliver Rev.

[24] Lee MH, Yang Z, Lim CW, Lee YH, Dongbang S, Kang C (2013). Disulfide-cleavage-triggered chemosensors and their biological applications. Chem Rev.

[25] Zhang R, Li Y, Hu B, Lu Z, Zhang J, Zhang X (2016). Traceable nanoparticle delivery of small interfering RNA and retinoic acid with temporally release ability to control neural stem cell differentiation for Alzheimer's disease therapy. Adv Mater.

[26] Wang Z, Zhang R, Wang Z, Wang HF, Wang Y, Zhao J (2014). Bioinspired nanocomplex for spatiotemporal imaging of sequential mRNA expression in differentiating neural stem cells. ACS Nano.

[27] Stolt CC, Lommes P, Sock E, Chaboissier MC, Schedl A, Wegner M (2003). The Sox9 transcription factor determines glial fate choice in the developing spinal cord. Gene Dev.

[28] Solanki A, Shah S, Yin PT, Lee KB (2013). Nanotopography-mediated reverse uptake for siRNA delivery into neural stem cells to enhance neuronal differentiation. Sci Rep.

[29] Grabar KC, Freeman RG, Hommer MB, Natan MJ (1995). Preparation and characterization of Au colloid monolayers. Anal Chem.

