# Supplementary material for: RNA-silencing nanoprobes for effective activation and dynamic imaging of neural stem cell differentiation
Source: Theranostics. 2019 Jul 9;9(18):5386–95. doi: 10.7150/thno.35032 (PMC6691577; doi:10.7150/thno.35032)
Supplement: Supplementary file 1 — Supplementary experimental procedures, figures and table. [file thnov09p5386s1.pdf]

Supporting Information

RNA-silencing nanoprobes for effective activation and dynamic imaging of neural stem cells differentiation

Ruili Zhang, Zhe Wang, Zuo Yang, Linlin Wang, Zhongdi Wang, Baihang Chen, Zhongliang Wang*,and Jie Tian*

Experimental Procedures

1. Loading Determination of DNA and siSOX9.

The concentration of cvNCs was determined by using UV-Vis absorption spectroscopy (λ = 525 nm, $\varepsilon = 3.64 \times 10^8 M^{-1}cm^{-1}$). DNA oligonucleotides were set free from cvNCs by dissolving their gold backbones in KCN solution of 0.1 M. The concentration of DNA duplexes was determined by fluorescence measurements and comparing to a standard curve (Figure S2). The loading number of DNA oligonucleotides and siSOX9 was calculated by dividing the concentration of dye-containing oligonucleotides by the concentration of gold colloidal nanoparticles.

2. Cytotoxicity of cvNCs.

The cytotoxicity of cvNCs to neural stem cells (NSCs) was evaluated by MTT assay. In MTT assay, 100 μL complete culture medium containing 5000 NSCs were cultured in a 96-well plate for 24 h before experiment. Various concentrations of cvNCs were incubated with cells for 48 h. The medium was replaced with 100 μL fresh media containing 10 μL of MTT solution (5 mg/mL), followed by incubation for another 4 h. The media was then removed, and 100 μL of dimethyl sulfoxide (DMSO) was added into each well to dissolve the formed formazan crystals. The absorbance at 570 nm was recorded using a microplate reader. The absorbance from the control cells was set as 100% cell viability (n=6).

3. Western Blot Study of βIII Tubulin and NeuN

NSCs were incubated with cvNCs, cvNCs with scrambled sequence, or non-cvNCs at predetermined time points, respectively. Then, cells were washed with ice cold PBS (calcium and magnesium free) three times before being treated with RIPA buffer (Pirece) with protease inhibitor cocktail (Roche) for 10 min on ice. Cell lysates were harvested and subject to centrifuge at 13000 rpm at 4 ºC for 10 min. The resultant supernatants containing protein were harvested, followed by 4%-12% gradient SDS-PAGE gel separation. Nitrocellulose membranes were used for transferring separated protein bands. The membranes were blocked by 5% skim milk for 1 h at room temperature, and then immunoblotted with anti-βIII tubulin (1:1000) and anti-NeuN (1:1000) primary monoclonal antibodies (Cell Signaling Technology, Inc.). HRP conjugated goat anti rabbit secondary antibody (1:3000) was used to visualize immunoreactive bands. Anti β-actin rabbit mAb (Cell Signaling Technology, Inc.)(1:2000) was used as the house keeping control.

4. Hybridization Experiment.

For multiplex analyst detection, Tubb3-targeted or Fox3-targeted DNA-Au NPs of 1 nM in PBS buffer was treated with the Tubb3 targets (single-base mismatched or complementary DNA targets) or Fox3 targets (single-base mismatched or complementary DNA targets), respectively at various concentrations of 0, 2, 5, 10, 25, 50, 100, 200 nM. After incubation for 1 h at 37 °C, the fluorescence was observed at appropriate excitation wavelengths. The fluorescence of Alexa 488 was excited at 490 nm and measured at 530 nm (range from 500 to 700 nm) and the fluorescence of Cy3 was excited at 550 nm and measured at 570 nm (range from 550 to 700 nm). All experiments were repeated at least three times.

Table S1. Gene sequences used in this study

Names of DNA	Sequences
Tubb3 recognizing sequence	5'-NH_2 mUmCmC mAAG TCC ACC AGA ATmG mGmCmC AAA AAA-dithiol-3'
Tubb3 Reporter	5'-Alexa488-TGG CCA TTC TGG-3'
Tubb3 DNA target	5'-GGC CAT TCT GGT GGA CTT GGA-3'
Tubb3 DNA target with one mismatch	5'-GGC CAT TCT G*C*T GGA CTT GGA-3'
Fox3 recognizing sequence	5'-NH_2 mCmAmU mUTT AAC AAG CGT TTmG mCmUmC AAA AAA-dithiol-3'
Fox3 reporter	5'-Cy3-TGA GCA AAC GCT-3'
Fox3 DNA target	5'-GAG CAA ACG CTT GTT AAA ATG-3'
Fox3 DNA target with one mismatch	5'-GAG CAA ACG C*A*T GTT AAA ATG-3'
siSOX9 antisense	5'-mAmAmC mGmAmG mAmGmC mGmAmG mAmAmG mAmGmA mCmCmC -NH_2-3'
siSOX9 sense	5'-mGmGmG mUmCmU mCmUmU mCmUmC mGmCmU mCmUmC mGmUmU mCmAmG-3'
Control *siSOX9* antisense	5'-mAmAmC m*A*mAmG mAm*C*mC m*A*mAm*C* m*G*mAmG mAm*A*mA mCm*A*mC-NH_2-3'
Cy5-*siSOX9* antisense	5'-mAmAmC mGmAmG mAmGmC mGmAmG mAmAmG mAmGmA mCmCmC -Cy5-NH_2-3'
Tubb3-siSOX9 sequence	5'-mGmGmG mUmCmU mCmUmU mCmUmC mGmCmU mCmUmC mGmUmU mCmAmG TTT TTT mUmCmC mAAG TCC ACC AGA ATmG mGmCmC AAA AAA-dithiol-3'
Fox3-siSOX9 sequence	5'-mGmGmG mUmCmU mCmUmU mCmUmC mGmCmU mCmUmC mGmUmU mCmAmG TTT TTT mCmAmU mUTT AAC AAG CGT TTmG mCmUmC AAA AAA-dithiol-3'
Survivin	5'-CAAGGAGCTGGAAGGCTG-3'
β-actin	5'-GCTACAGCTTCACCACCACAG-3'
Nestin	5'-GTCTCAGGACAGTGCTGAGCCTTC-3'
GAPDH	5'-GGTCTCCTCTGACTTCAACA-3'
CXCR4	5'-CGGCAGCAGGTAGCAAAGTGAC-3'

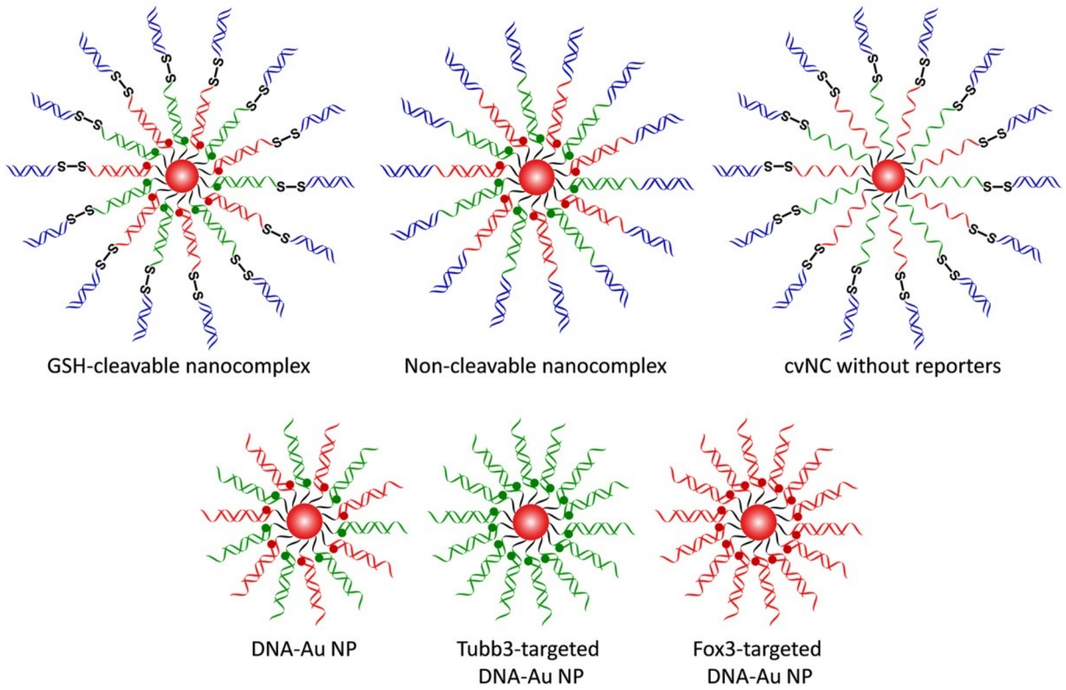

Figure S1. Schematic representation describing the structures of GSH-cleavable nanocomplex (cvNC), non-cleavable nanocomplex (non-cvNC), cvNC without reporters, DNA-Au NP, Tubb3-targeted DNA-Au NP, and Fox3-targeted DNA-Au NP.

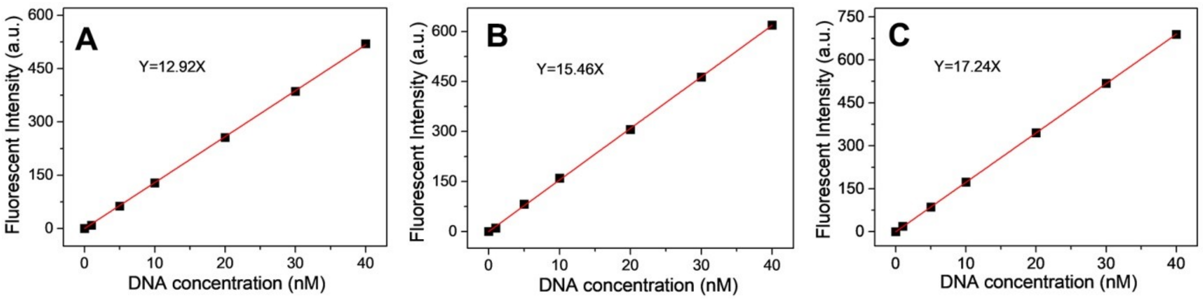

Figure S2. Calibration curves. Plot of fluorescence intensity as the function of DNA concentration: (A) Alexa 488-modified Tubb3 oligo, (B) Cy3-modified Fox3 oligo, and (C) Cy5-modified siSOX9.

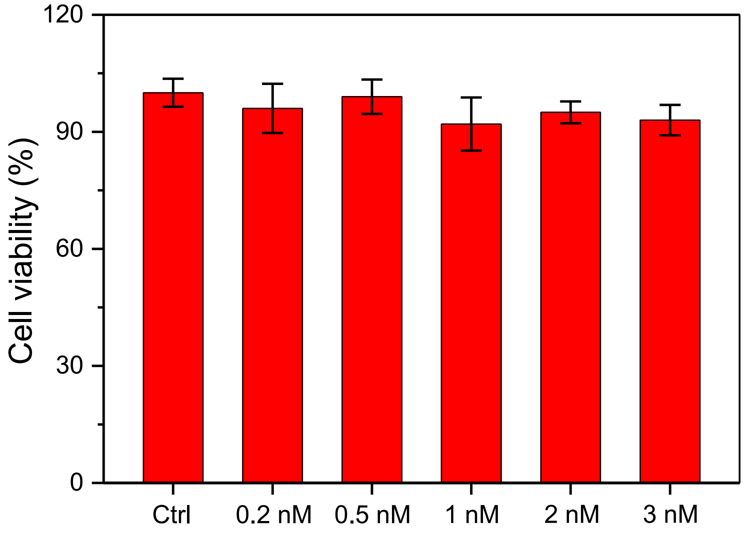

Figure S3. Cytotoxicity assays of cvNCs to neural stem cells with MTT assay.

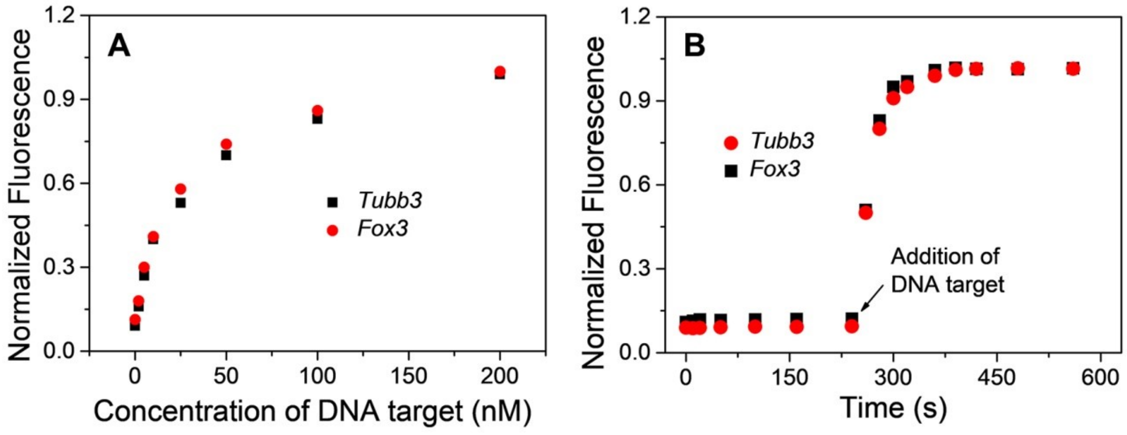

Figure S4. (A) Dynamic response of *Tubb3*-targeted DNA-Au NPs or *Fox3*-targeted DNA-Au NPs to corresponding DNA targets with various concentrations of 0, 2, 5, 10, 25, 50, 100, and 200 nM. (B) Hybridization kinetics of *Tubb3*-targeted DNA-Au NPs or *Fox3*-targeted DNA-Au NPs associated with the complementary DNA targets.

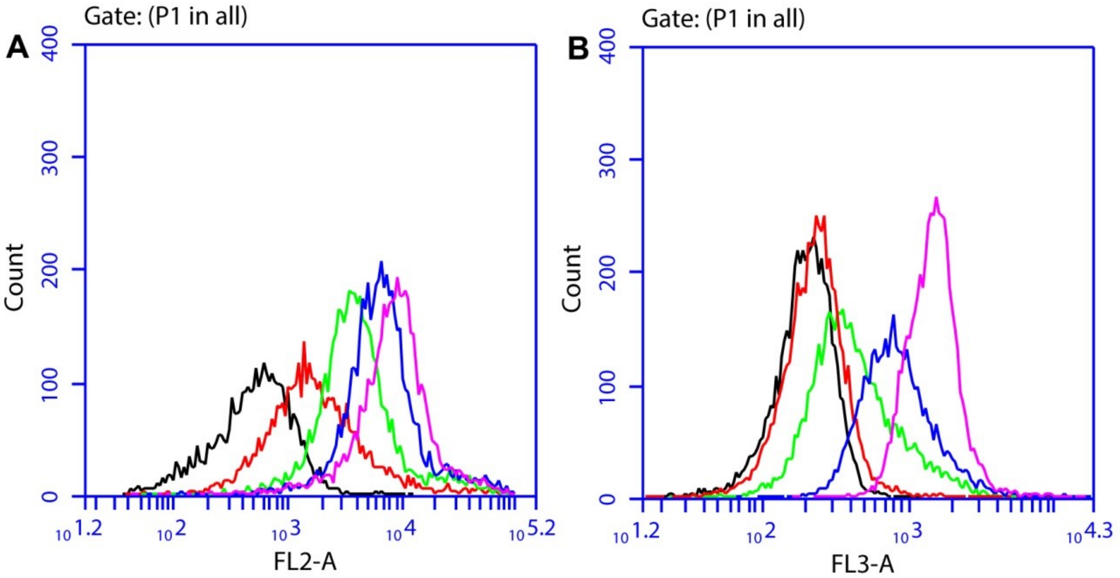

Figure S5. Flow cytometry results for (A) *Tubb3* and (B) *Fox3* of cvNCs-treated NSCs at different incubation time points. Black: non-treatment; Red: 3 days; Green: 5 days; Blue: 8 days; and Purple: 10 days.

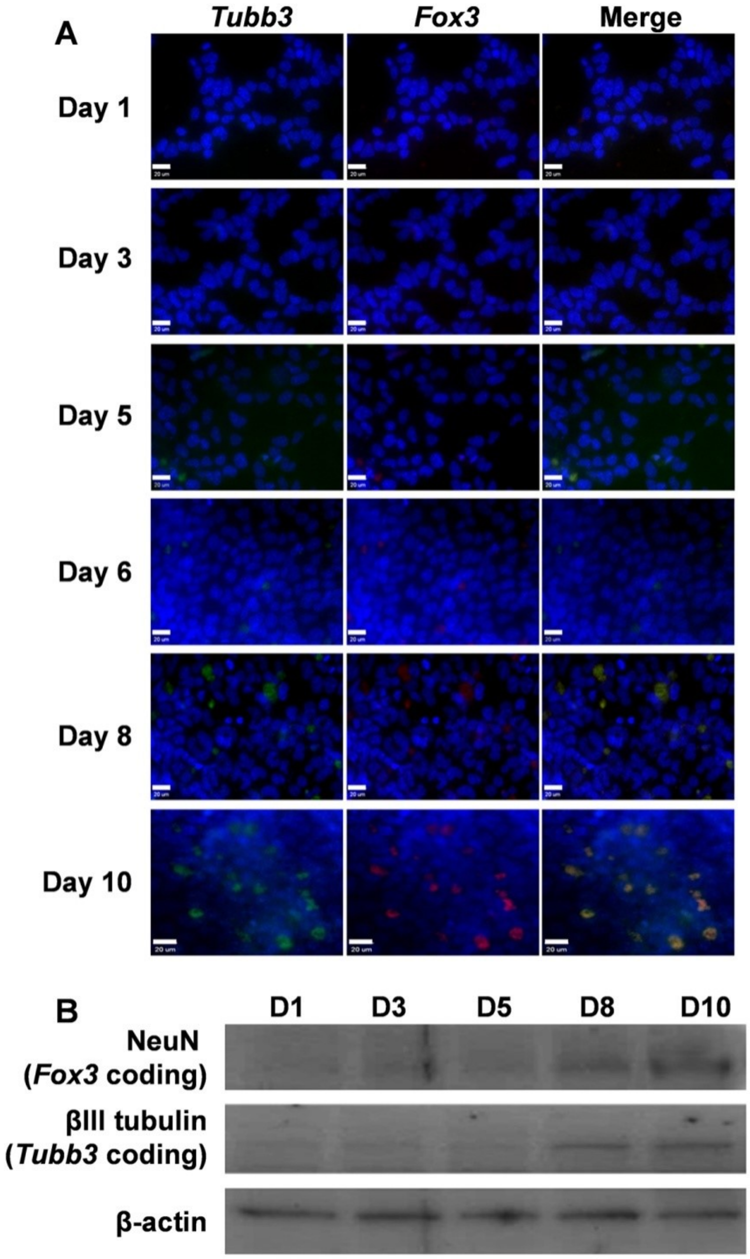

Figure S6. Negative control of non-cvNCs for real-time imaging of *Tubb3* and *Fox3* mRNA expressions over 10 days. Blue: Hoechst 33342. Green: *Tubb3* mRNA; red: *Fox3* mRNA. Scale = 20 μm.

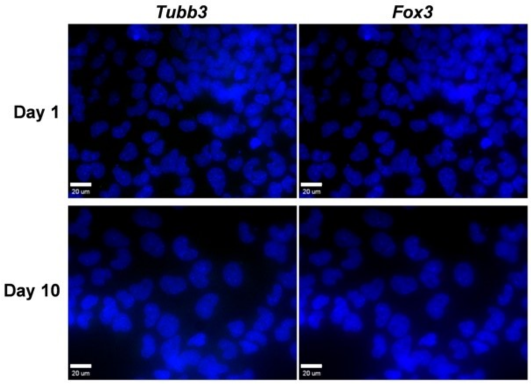

Figure S7. Negative control of cvNCs with scrambled sequence for real-time imaging of *Tubb3* and *Fox3* mRNA expressions over 10 days. Blue: Hoechst 33342. Green: *Tubb3* mRNA; red: *Fox3* mRNA. Scale = 20 μm.
